# Local decomposition induced by dislocation motions inside tetragonal Al_2_Cu compound: slip system-dependent dynamics

**DOI:** 10.1038/srep03157

**Published:** 2013-11-07

**Authors:** D. Chen, X. L. Ma

**Affiliations:** 1 Shenyang National Laboratory for Materials Science, Institute of Metal Research, Chinese Academy of Sciences, Wenhua Road 72, Shenyang 110016, China

## Abstract

Dislocations in a crystal are usually classified into several independent slip systems. Motion of a partial dislocation in monometallic crystals may remove or create stacking fault characterized with a partial of a lattice translation vector. However, it is recently known that motion of partial dislocations in complex structure, such as that inside an intermetallic Al_2_Cu compound, lead to a local composition deviation from its stoichiometric ratio and the resultant structure collapse. Here we report such a local decomposition behaviors are strongly dependent on slip system of dislocations. Under applied external stress, we have studied dislocation motion behaviors in the three independent slip systems of [001](110), [100](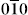) and [110](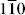) within tetragonal Al_2_Cu crystal by using molecular dynamics method. We found dislocation motions in all these slip systems result in local decomposition but their physical details differ significantly.

Relationship between microstructure and properties is the key issue in materials science. Dispersion strengthening is of great importance in enhancing mechanical properties of engineering alloys, in which small particles of second phase (usually with complex crystal structure) play a critical role in resisting dislocation motion in the matrix. Al2Cu intermetallic particles offer dispersion strengthening in Al-Cu alloys in which the presence of Al_2_Cu is able to block dislocations motion and consequently harden the materials^1^,^2^,^3^,^4^,^5^. Therefore, the higher concentration of Al_2_Cu particles offer, the higher hardness of the alloy has. However, in the past decades, more and more experimental observations indicate that the small-sized second phase can be decomposed during plastic deformation, leading to degradation of material properties. Such a decomposition of the strengthening particles under mechanical loading may unavoidably lead to a detrimental consequence. The intrinsic mechanism of deformation-induced destabilization of complex compounds in some materials has been debated for decades^6^,^7^,^8^,^9^, in which dislocation behaviors received great concerns.

A classical description on dislocations was that their motions in a crystal do not induce any compositional change in the area they pass by, and moreover, most of dislocation theory was developed for dislocations in continuous media and atomic nature of the medium was not taken into account. However, dislocations in complex solids are much more complicated as compared with those in simple monometallic crystals, since the core structure of dislocations and their motion behaviors are strongly associated with crystal structure of the medium^10^.

With the development of high-resolution transmission electron microscopic (HRTEM) imaging combined with theoretical calculation, it has become possible to identify dislocation core details in a particular structure. Nevertheless, the underlying mechanism on dislocation motion-induced phase local decomposition inside precipitates is rarely explored^2^,^3^. Recently, we investigated the dislocations details inside the Al_2_Cu precipitates in an Al-alloy, and we focused on the dislocation motion behaviors under applied external stress. We found that the operation of [001](110) slip system would induce a local phase decomposition in such a complex structure in a particular manner^11^. Dislocations in a crystal are usually classified into several slip systems. The gliding of dislocations has its particular characteristics which depend on slip systems. The objective of the present study is to figure out, at atomic scale, the details and dynamics of edge-type dislocations on three independent slip systems of [001](110), [100](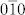) and [110](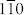) in tetragonal Al_2_Cu (for the sake of convenience, these dislocations are respectively labeled as Dislocations I, II and III) based on the experimental results^5^,^11^,^12^. This work is expected to provide new information on dislocation motion behaviors in complex structures.

## Results

In our experimental studies, dislocations associated with the slip system of [001](110) are frequently observed inside the Al_2_Cu precipitates. High resolution imaging demonstrates that Dislocation I is usually dissociated into two partials with a stacking fault in between. For each partial, a lattice displacement of 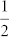
[001] can be identified^11^. Accordingly, a computational cell including two partials was constructed in which the observed extended dislocation configurations are embedded, and the equilibrium separation distance of the two partials is about 14.4 nm after optimization falling within the range of experimental observation (10 ~ 20 nm)^11^.

**Figure 1:**
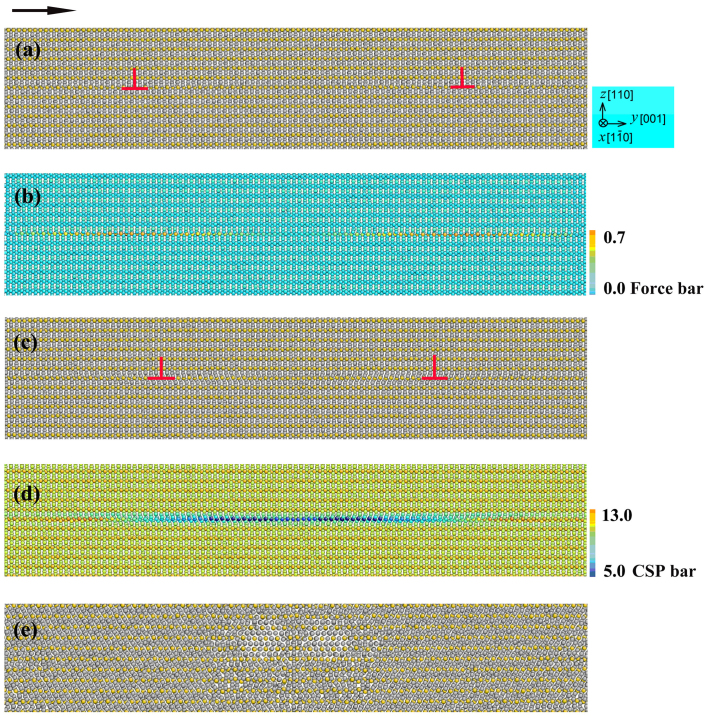
Snapshots of the atomic configuration with Dislocation I projected on the (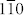) plane (a, b) after relaxation, and at (c, d) 9,000 and (e) 75,000 timesteps. In (a, c, e), the gray spheres are Al atoms and the yellow spheres are Cu atoms, the black arrow shows the direction of Top atomic velocity. In (b), the atoms are colored by the force on them along the horizontal ([001]) direction (*y* axis) ranging from 0.0 eV/Å to 0.7 eV/Å. In (d), the atoms are colored by the values of centrosymmetry parameters (CSP) ranging from 13.0 Å^2^ to 5.0 Å^2^.

**Figure 2:**
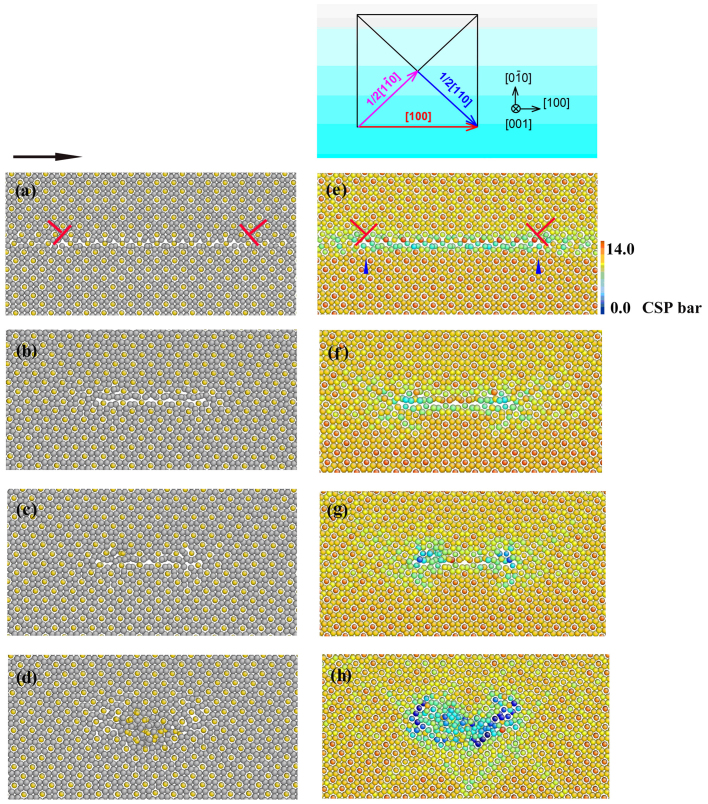
Snapshots of the atomic configuration with Dislocation II projected on the (001) plane (a, e) after relaxation, and at (b, f) 55,000, (c, g) 75,000 and (d, h) 130,000 timesteps. In (a–d), the gray spheres are Al atoms and the yellow spheres are Cu atoms, the black arrow shows the direction of Top atomic velocity. In (e–h), the atoms are colored by the values of centrosymmetry parameters (CSP) ranging from 14.0 Å^2^ to 0.0 Å^2^. The inset of Fig. 3 is depicted as an illustration for the Burgers vectors.

Figures 1 (a) and (b) show the atomic configurations with the left and right partials after relaxation projected on the (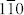) plane. It should be noted that the partials are two different kinds of atomic arrangement configurations because the spatial atomic arrangement of each core is quite different along the [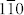] direction. In Fig. 1(b), the atoms are colored by the force on them along the horizontal ([001]) direction (*y* axis) ranging from 0.0 eV/Å to 0.7 eV/Å (1 eV/Å = 1.603 × 10^−9^ N), which indicates that each partial is forced at its core along the (110) plane as soon as the simulation cell is applied by an external stress. Figs. 1(c) and 2(d) show the snapshot of atomic configuration at 6,000 timesteps, which illustrate that each partial can be activated and move on the (110) plane along the [001] direction. In the meantime, the two partials tend to contract under applied external stress. The contraction of extended dislocations might lower their kinetic energies enough to offset the stacking fault energies that determine their extensions, as proposed theoretically for fcc crystals^13^. In Fig. 1(d), the atoms are colored according to the values of the centrosymmetry parameters^14^, by which local state in the vicinity of a given atom can be quantified. It is seen that, in Fig. 1(d), there is a tendency of local distortions on the gliding plane at this moment. That is to say, the motion of each partial is essentially a migration of a group of special atomic arrangement configuration with local distortions.

Furthermore, the development of the partial motion makes the field expansion of local distortions, which corresponds to changing the partial motion state: each partial moves more and more roughly. Eventually, the expansion of local distortions takes the place of the partials. Fig. 1(e) demonstrates the snapshot of atomic configuration at 75,000 timesteps where partials cannot be detected any longer. This reveals that the dislocation motions in [001](110) slip system of Al_2_Cu may lead to chemical decomposition and structural collapse around slip planes.

Dislocation II can also be dissociated into two partials with a stacking fault in between. Figure 2 shows the snapshots of atomic configuration of Dislocation II. In Fig. 2(a–d), the gray spheres are Al atoms and the yellow spheres are Cu atoms. In Fig. 2(e–h), the atoms are colored by the values of centrosymmetry parameters. Figures 2(a) and 2(e) demonstrate the atomic configuration of Dislocation II after relaxation projected on the (001) plane, in which the dislocation (Burgers vector [100]) splits into two partials (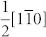] and 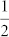
[110]), the distance between partials (stacking fault width) is about 6.0 nm. The inset of Fig. 2 is depicted as an illustration for the Burgers vectors. The two partials seem to be activated at the beginning when the external stress is applied. With the development of simulation, the distance between partials shrinks on the (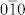) plane since they move in opposite direction towards the original center of the stacking fault. Figs. 2(b) and 2(f) exhibit configurations at 55,000 timesteps where we can see a reduced distance of two partials and the resultant disordered structures. When the partials further contract, the disorders are more remarkable as shown in Figs. 2(c) and 2(g) (at 75,000 timesteps). At 130,000 timesteps, a notable region of disorder is formed as shown in Figs. 2(d) and 2(h). Upon the further application of external stress, the disordered region spreads more till to 300,000 timesteps.

**Figure 3:**
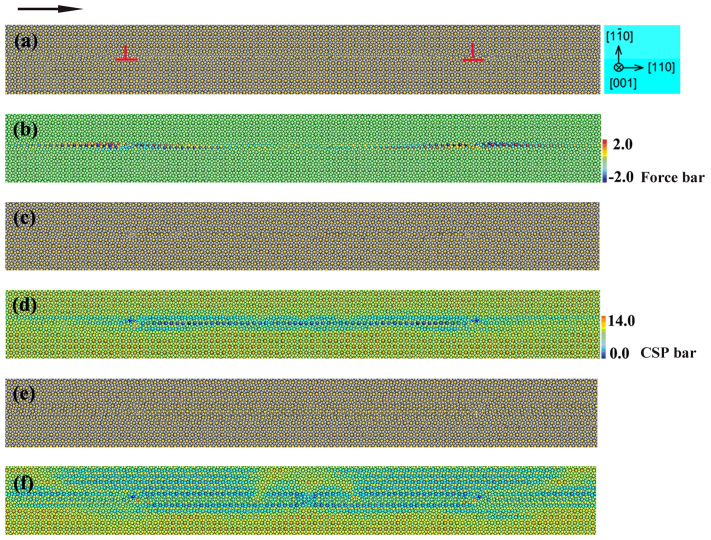
Snapshots of the atomic configuration with Dislocation III projected on the (001) plane (a, b) after relaxation, and at (c, d) 6,000 and (e, f) 20,000 timesteps. In (a, c and e), the gray spheres are Al atoms and the yellow spheres are Cu atoms, the black arrow shows the direction of Top atomic velocity. In (b), the atoms are colored by the force on them along the horizontal ([110]) direction (*y* axis) ranging from −2.0 eV/Å to 2.0 eV/Å. In (d, f), the atoms are colored by the values of centrosymmetry parameters (CSP) ranging from 14.0 Å^2^ to 0.0 Å^2^.

Dislocation III can be dissociated into two partials with a stacking fault in between, too. For each partial, the Burgers vector is 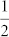
[110]. Figure 3 shows the snapshots of atomic configuration of Dislocation III. In Figs. 3(a), 3(c) and 3(e), the gray spheres are Al atoms and the yellow spheres are Cu atoms. In Fig. 3(d) and 3(f), the atoms are colored according to the values of centrosymmetry parameters. Figure 3(a) and 3(b) show the atomic configurations after relaxation projected on the (001) plane, in which the equilibrium separation distance of the two partials is about 24.5 nm. In Fig. 3(b), the atoms are colored by the force on them along the horizontal ([110]) direction (*y* axis) ranging from −2.0 eV/Å to 2.0 eV/Å (the value is positive if the force is along the positive direction of the *y* axis referring to the coordinate system shown in Fig. S1), which illustrate that the configuration of each partial is asymmetric due to the essential asymmetry of the core structure of 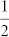
[110] dislocation.

Under applied external stress, each partial is inert to move which results in structural disorders. Figs. 3(c) and 3(d) display the snapshot of each inactive partial with a state of disorders at 6,000 timesteps. Each partial seems to be changed into a disorder region, where there is a great strain which may be responsible for the immobile effect. With the MD timesteps going, the region of atomic disorder spreads. At 20,000 timesteps, Figs. 3(e) and 3(f) show that the agglomerate of each disorder region is approximately around the original position of each partial. Such a disorder may finally spread to the whole range of computational cell with the continuous simulation.

Thus, the motion behaviors of Dislocation III are quite different from those of Dislocation I and Dislocation II. As we have seen, motion of Dislocation I may lead to the localized Cu-segregation around its slip plane. Under the influence of external stress, Dislocation I can slip and move on the glide plane,which leads to the breaking of the bonding structure at the core. The migration energy barrier of Cu (0.28 eV) is lower than that of Al (1.10 eV) and the atomic radius of Cu (1.28Å) is smaller than that of Al (1.43Å)^15^, as a sequence, Cu atoms would be more mobile and prefer to segregate which depends on the number of broken bonds in the local decomposition region. In contrast, Dislocations III and II share similar characteristics of local immobility. Under the application of external stress, these dislocations do not slip, but the core area gets distortions. That is to say, dislocation motions in all these slip systems could result in local decomposition but their physical details differ significantly.

## Discussion

The atomistic processes in MD simulations based on the three slip systems of tetragonal Al_2_Cu indicate that the local microstructural evolution is closely related to the atomic structure of dislocation core. In the Al_2_Cu compound, the effects of dislocation structures on their motion behaviors are very pronounced on the slip systems of [001](110), [100](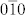) and [110](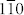). With the aid of external stress, the process of dislocation dynamics is in fact a transition of dislocation from its initial structure to a disordered structure. Such a process could be favored by the internal energy that is determined by the intrinsic property of dislocation behavior.

Generally, stability and internal energy are inversely proportional in a system, the lower the internal energy of the system, the more stable it is. The Gibbs free energy Δ*G* is related to enthalpy change Δ*H* by the Gibbs equation Δ*G* = Δ*H* − *T*Δ*S*, where *T* is the absolute temperature and Δ*S* is the entropy change. In our simulations, *T* is set as 0.1 K and the *T*Δ*S* becomes negligible when it is compared to Δ*H*. Therefore, Δ*H* can be used to show changes of internal energy in the present slip systems.

It is known that an accurate determination of the formation enthalpy of each dislocation is an important issue both in the experimental and theoretical communities. For the three systems of [001](110), [100](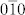) and [110](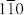), their calculated formation enthalpies are −24.436 KJ/mol, −22.904 KJ/mol and −20.731 KJ/mol, respectively. At the dislocation cores, the bonds between atoms are not in an equilibrium configuration, which is different from that in the perfect crystal, i.e. they are heavily distorted at their minimum enthalpy value. In this work, the enthalpy change of a slip system at any step is defined as the difference between the enthalpy at this moment and the enthalpy value at the beginning of the simulation process (i.e. the initial enthalpy of the system). Partial dislocations that bound the stacking faults in extended dislocations are the basic defect responsible for deformation mechanism in fcc metals^16^,^17^,^18^.

**Figure 4:**
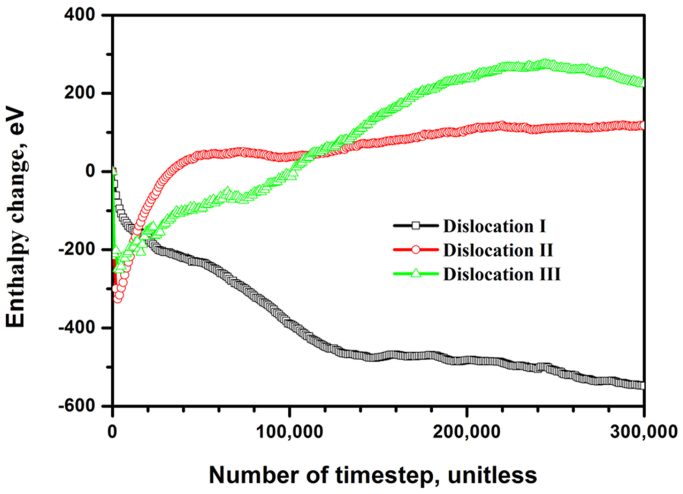
Enthalpy changes as a function of timesteps with different slip systems.

The behaviors of partial dislocations have to be taken into account in understanding the mechanisms of deformation-induced decomposition inside the Al_2_Cu precipitates in an Al-alloy. In the present study, the left partial (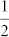
[001](110)) of Dislocation I and the left partial (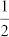
[110](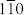)) of Dislocation III are chosen, respectively, as representative examples in this study, and the simulations with different slip systems are carried out until 300,000 timesteps. In contrast, for Dislocation II, the extended dislocation is considered since its width is much smaller than those of Dislocations I and III. Figure 4 plots the enthalpy changes as a function of timesteps with different slip systems. For the left partial of Dislocation I, the curve illustrates a pathway of energy change, all of which are negative. There is almost no energy barrier during the simulation process. For the segment of the curve from the beginning to about 10,000 timesteps, the slope is very steep which indicates that the partial is hardly activated on the (110) plane when an external stress is applied to the system. After that, the slope of curve has slowed down since the local distortions are produced on the slip plane. By the end of the simulation, the downward trend of curve reflects that the local decomposition is energetically preferred during the period in the system. In other words, phase decomposition is thermodynamically favorably in Dislocation I.

For Dislocations II and III, Fig. 4 shows different pathways of enthalpy changes. However, these two have something similar: both curves show a steady uptrend with a drawdown. When subjected to applied stress, the atoms in either system could be driven to form a temporary structure having the minimum levels of enthalpy changes at about 5,000 time steps, which is a very short duration of the event. Therefore, it might be not an absolute description that Dislocations II and III can not slip. Rather, they remain a temporary movement (slip) in these cases. After the minimum state, the curves begin to rise and the disorders are induced due to the resistance determined by the intrinsic property of dislocation itself. Since then, the atomic configurations in either system would contain a certain concentration of structural disorder around the dislocation core. At the end of simulations (300,000 time steps), the positive values of enthalpy change indicate non-favorable in energy. During the simulated process, the stress fields generated in the disorder regions interact with the applied external stress of each dislocation, thereby increasing the stress leads to lattice strain which can "anchor" dislocations, thus achieve a state of low potential energy. We emphasize that the data shown here are able to apply to the processes under applied external stress without conventional thermal activation.

This work presents the dynamic behaviors of dislocations on three slip systems of [001](110), [100](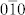) and [110](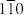) in the Al_2_Cu compound. The focus of the analyses is to demonstrate that the dislocation behaviors are closely bound up with dislocation structures, which are attributed an intrinsic property of each dislocation. The dislocation on the [001](110) system is activated on the (110) plane with local distortions once it has been applied by an external stress, which is a major cause of the later development of local decomposition. Although the behaviors of dislocations on the [100](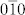) and [110](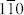) systems have difference pathways in the simulated processes, they are both inert to move which give rise to a disordered region with a great strain. The spatial coupling of dislocation slip and compound decomposition is of significance in advancing our understanding of the processing-property relationship in materials, and also in optimizing mechanical properties of engineering materials by means of controlled plastic deformation processing.

## Methods

To observe the atomic structural development in the Al_2_Cu compound under the application of external stress, a Large-scale Atomic/Molecular Massively Parallel Simulator (LAMMPS) is used to perform simulations throughout the work. Details on atomic scale simulations can be found in supplementary information.

## Author Contributions

This project was conceived by X.L. Ma; D. Chen carried out the theoretical calculations; All the authors participated in writing the paper.

## Supplementary Material

Supplementary Information Local decomposition induced by dislocation motions inside tetragonal Al2Cu compound: slip system-dependent dynamics
