# Supplementary material for: Local decomposition induced by dislocation motions inside tetragonal Al2Cu compound: slip system-dependent dynamics
Source: Sci Rep. 2013 Nov 7;3:3157. doi: 10.1038/srep03157 (PMC3819612; doi:10.1038/srep03157)
Supplement: Supplementary Information — Local decomposition induced by dislocation motions inside tetragonal Al2Cu compound: slip system-dependent dynamics [file srep03157-s1.pdf]

Supporting Online Materials for

Local decomposition induced by dislocation motions inside tetragonal Al_2Cu compound: slip system-dependent dynamics

D. Chen and X. L. Ma*

Shenyang National Laboratory for Materials Science, Institute of Metal Research, Chinese Academy of Sciences, Wenhua Road 72, Shenyang 110016, China

* Correspondence and request should be addressed to X.L. Ma (xlma@imr.ac.cn)

Methods

Atomic scale simulations

In our experimental study, dislocations with Burgers vectors of [001] and [100] were frequently observed on (110) and (001) planes[1], and dislocations with [110]{110} were also reported[2-3]. Based on these experimental results[1-3], we employ the dislocations on the three slip systems of [001](110), $[100](0\bar{1}0)$ and $[110](1\bar{1}0)$ to study the response of each dislocation to external stress at a microscopic level. In sequential order, for the sake of convenience, these dislocations are labeled as Dislocations I, II and III, respectively, in the work.

To observe the atomic structural development in the Al_2Cu compound under the application of external stress, a Large-scale Atomic/Molecular Massively Parallel Simulator (LAMMPS) is used to perform simulations throughout the work. The MD simulations based on the embedded atom method (EAM) potential were employed to study the dislocation behaviors and understand the mechanism of deformation-induced decomposition inside the Al_2Cu precipitates. The characteristic mechanisms favor the molecular models without thermal effect, because reducing thermal effect is one of the major concerns in experiments. Dislocations in our earlier experiments were introduced at liquid nitrogen temperature (~70 K) by using the surface mechanical attrition treatment (SMAT), which produces a gradient variation of plastic strain from a high value to zero within the thin surface layer[1]. For this purpose, we need a reference from quantum mechanics first-principles calculations for each case. Our simulations were first performed by using two different EAM interatomic potentials [4-5] for the properties of bulk Al_2Cu at 0.1 K. As is well known, the EAM potentials implicitly include thermal effects at the temperature of the experimental data measurement because they are usually

constructed by fitting to experimental data[6-7]. Our calculated formation enthalpy (-18.8 KJ/mol) based on the potential of Cai *et al*[5] is in good agreement with the first-principles results (-16.3 KJ/mol[8], -17.8 KJ/mol[9]) compared with that (-10.9 KJ/mol) based on Liu's potential[4], which indicates the reliability of the interatomic potential[5] close to absolute zero. In choosing EAM potentials, our criterion is that they were well or best described at zero degree Kelvin. In the work, calculations based on the potential of Cai *et al*[5] can include anyway for completeness and provide valuable information on the mechanical and dynamical properties near absolute zero in temperature.

Figure S1 is the schematic diagram of simulation cell, in which the initial structure of edge dislocation is created by removing two adjacent atomic half planes from the crystal structure. The geometry in the simulation cell contains a dislocation along the x axis and has periodic boundaries in the direction of dislocation line, with fixed boundaries along y and z axes. For Dislocation I, the *x* axis is along the $[1\bar{1}0]$ direction, the *y* axis along the [001] direction and the *z* axis along the [110] direction. Dislocations I, II and III are constructed with 22912 atoms (with an edge size of 17.2, 289.9 and 72.6 Å along the x, y and z axes), 8496 atoms (with a volume of 4.9×178.8×76.5 $Å^3$) and 21408 atoms (9.8×510.2×68.3 $Å^3$), respectively. In Fig. S1, Top atoms are given a set velocity but Bottom atoms are fixed. In experiments, SMAT can produce a gradient variation of plastic strain (and strain rate) from a high value to zero within the thin surface layer. By this method, the strain rate induced was as high as $10^3 \sim 10^5$ s^{-1} in the top layer[10,11], in which more volume of Al_2Cu particles was reduced. Al_2Cu particles, which are away from the topmost layer (to say, about 40 μm thick from the top), experienced less strain during SMAT and in such a case Al_2Cu remains coarse grain size but local decomposition occurring along the slip plane can be well recorded[1]. Accordingly the simulations were performed to a computational cell in which the Top atoms are given a set velocity (v_{top}) of 0.1 m/s along y axis. Such a velocity to the structure corresponds to a strain rate of about $5.79\times10^4 s^{-1}$, $1.56\times10^5 s^{-1}$ and $6.15\times10^4 s^{-1}$ for Dislocations I, II and III respectively (comparable to those in experiments). In the system, the generated stress propagates from Top to the dislocation on the central plane. The total energy minimization criterion, which is based on the conjugate gradient algorithm, is used to optimize the atomic structure and relax the system to equilibrium. In addition to monitor the atomistic processes during dislocation motion, we also applied the centrosymmetry parameter to analyze the large amount of data in such atomistic simulations. The centrosymmetry parameter is a measure of the local lattice disorder around an atom and can be used to characterize

whether the atom is part of a perfect lattice, or a local defect (a dislocation or stacking fault) in solid state systems[1,12].

References

1. Yang, B., Zhou, Y. T., Chen, D., Ma, X. L. Local decomposition induced by dislocation motions inside precipitates in an Al-alloy. *Sci. Rep.* **3**, 1039 (2013).
2. Galy, D., Boulanger, L. Transmission electron-microscopy study of plastic-deformation in $NiZr_2$. *J. Mater. Sci.* **30**, 1766-1771 (1995).
3. Bonnet, R., Loubradou, M. Crystalline defects in a BCT $Al_2Cu(\theta)$ single crystal obtained by unidirectional solidification along [001]. *Phys. Stat. Sol. (a)* **194**, 173-191 (2002).
4. Liu, X. Y., Liu, C. L., Borucki, L. J. A new investigation of copper's role in enhancing Al-Cu interconnect electromigration resistance from an atomistic view. *Acta Mater.* **47**, 3227-3231 (1999).
5. Cai J., Ye, Y. Y. Simple analytical embedded-atom-potential model including a long-range force for fcc metals and their alloys. *Phys. Rev. B* **54**, 8398-8410 (1996).
6. Williams, P. L., Mishin, Y., Hamilton, J. C. An embedded-atom potential for the Cu-Ag system. *Modelling Simul. Mater. Sci. Eng.* **14**, 817-833 (2006).
7. Karimi, M., Stapay, G., Kaplan, T., Mostoller, M. Temperature dependence of the elastic constants of Ni: reliability of EAM in predicting thermal properties. *Modelling Simul. Mater. Sci. Eng.* **5**, 337-346 (1997).
8. Zhou, W., Liu, L. J., Li, B. L., Song, Q. G., Wu, P. Structural, elastic, and electronic properties of Al-Cu intermetallics from first-principles calculations. *J. Electron. Mater.* **38**, 356-364 (2009).
9. Wolverton, C., Ozoliņš, V. Entropically favored ordering: The metallurgy of Al_2Cu revisited. *Phys. Rev. Lett.* **86**, 5518-5521 (2001).
10. Chan, H. L., Ruan, H. H., Chen, A. Y., Lu, J. Optimization of the strain rate to achieve exceptional mechanical properties of 304 stainless steel using high speed ultrasonic surface mechanical attrition treatment. *Acta Mater*. **58**, 5086-5096 (2010).
11. Zhang, X. C., Lu, J., Shi, S. Q. A computational study of plastic deformation in AISI 304 induced by surface mechanical attrition treatment. *Mech. Adv. Mater. Struc.* **18**, 572-577 (2011).
12. Kelchner, C. L., Plimptom, S. J., Hamilton, J. C. Dislocation nucleation and defect structure during surface indentation. *Phys. Rev. B* **58**, 11085-11088 (1998).

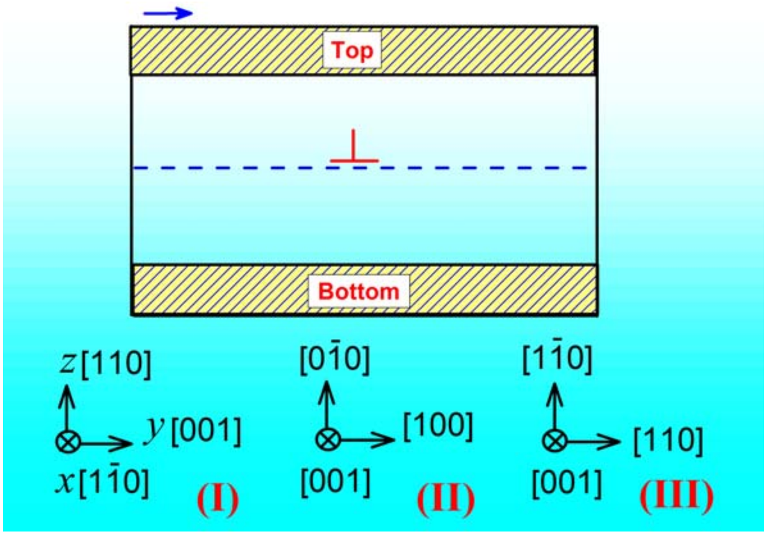

Fig.S1. Schematic of a computational cell used for MD simulations (Fig. 4(a) in Ref. 1). The dislocation is indicated by red ⊥, the blue arrow shows the direction of Top atomic velocity. Top atoms are allowed to move only horizontally along the *y* direction, while Bottom atoms are fixed. The insets of Fig. 1 show the coordinate systems for Dislocations I, II and III, respectively. Crystallographic directions are indicated by ⊗ and the arrows.
